# Mammalian Target of Rapamycin Signaling in the Spinal Cord Is Required for Neuronal Plasticity and Behavioral Hypersensitivity Associated With Neuropathy in the Rat

**DOI:** 10.1016/j.jpain.2010.03.013

**Published:** 2010-12

**Authors:** Curtis O. Asante, Victoria C. Wallace, Anthony H. Dickenson

**Affiliations:** Department of Neuroscience, Physiology and Pharmacology, University College London, London, UK

**Keywords:** Rapamycin, spinal, hypersensitivity, neuropathic, injury

## Abstract

The protein kinase mammalian target of rapamycin (mTOR) regulates mRNA translation and is inhibited by rapamycin. Signaling pathways involving mTOR are implicated in physiological and pathophysiological processes. We determined the spinal effects of the rapamycin analogue cell cycle inhibitor (CCI)-779 on neuronal responses and behavioral hypersensitivity in a model of persistent neuropathic pain. We also assessed the anatomical distribution of spinal mTOR signaling pathways. Specifically, we ligated rat spinal nerves L5 and L6 to produce a model of neuropathic pain. After confirming neuropathy with behavioral testing, we obtained in vivo single-unit extracellular stimulus-evoked recordings from deep dorsal horn spinal neurons. We applied CCI-779 spinally in electrophysiological and behavioral studies and assessed its effects accordingly. We also used immunohistochemistry to probe for mTOR signaling pathways in dorsal root ganglia (DRG) and the spinal cord. We found that spinally administered CCI-779 rapidly attenuated calibrated mechanically but not thermally evoked neuronal responses and mechanically evoked behavioral responses. Immunohistochemistry showed presence of mTOR signaling pathways in nociceptive-specific C-fiber DRG and in neurons of inner lamina II of the spinal cord. We conclude that alterations in the activity of spinal mTOR signaling pathways are crucial to the full establishment of spinal neuronal plasticity and behavioral hypersensitivity associated with nerve injury.

**Perspective:**

This study is consistent with growing evidence implicating mTOR signaling pathways as important modulators of persistent pain, providing novel insights into the molecular mechanisms of pain maintenance and potential for novel approaches into treating chronic pain.

Neuropathic pain is a complex disorder that can become chronic and severely impair an individual's quality of life. Despite an increase in basic and clinical knowledge, the current available treatment for neuropathic pain treatment remains inadequate^2^ and the search continues not only for improved therapies, but also for novel targets.

Local mRNA translation is a mechanism by which cells, and, indeed, neurons can supply proteins to specific locations. The mammalian target of rapamycin (mTOR), a serine-threonine protein kinase, which is inhibited by the immunosuppressant drug rapamycin, regulates several intracellular pathways in response to various extracellular signals and thereby regulates mRNA translation. These pathways involve mTOR-dependent activation of the 70 kDa ribosomal protein S6 kinase (p70S6K) as well as the inactivation of the repressor of mRNA translation, eukaryotic initiation factor 4E (eIF4E) binding protein (4EBP).^4,8^ It is perhaps not surprising that mTOR activity is modified in a wide range of pathological states, such as cancer, and neurodegenerative disorders, such as Alzheimer's disease.^11,34^

A range of interesting studies has shed light on the roles of mTOR signaling pathways in neuronal excitability. mTOR signaling pathways have been shown to be important in injury-induced hyperexcitability of *Aplysia* axons^39^ and play time-restricted roles in hippocampal long-term potentiation (LTP).^5^ Perhaps more relevant, local peripheral mTOR signaling pathways have been shown to play a major role in chronic nerve injury, since peripherally applied rapamycin attenuates fully established nerve injury-induced hypersensitivity.^12^ These studies reveal insights into the possible roles these mechanisms play in the peripheral and central nervous systems and, more specifically, chronic pain syndromes. Our studies focus on central mTOR signaling pathways in the spinal cord and their role in nerve injury-induced neuronal and behavioral changes, an area that as well as peripheral mTOR signaling pathways has gained much interest in recent times (see Price and Geranton^26^ for review)

Kim et al^14^ have shown that spinal protein synthesis is an important component of the behavioral hypersensitivity induced by injection of formalin into the hind paw of mice. In addition, Price et al^27^ have implicated specific spinal mRNA translation pathways in formalin-induced behavioral hypersensitivity. Their studies used mice lacking fragile X mental retardation gene (fmr1), another protein influencing mRNA translation. fmr1 knockout mice were shown to display reduced formalin-induced behavioral hypersensitivity compared to their wild-type littermates. Furthermore, spinal administration of rapamycin was ineffective in attenuating formalin-induced behavioral hypersensitivity in fmr1 mutant mice compared to their wild-type littermates, showing that mTOR signaling pathways modulate persistent painlike states as well as interact with other mRNA translation pathways. Interestingly, Codeluppi et al^6^ have identified a role for nonneuronal spinal mTOR signaling pathways in spinal cord injury in the context of spinal cord injury. Most recently, Geranton et al^7^ have shown that spinal nerve injury-induced behavioral hypersensitivity can also be attenuated by spinal administration of rapamycin.

We have recently shown that inhibiting mTOR signaling pathways prior to the formalin response attenuates acute behavioral hypersensitivity and neuronal hyperexcitability.^1^ In this present study, we inhibited spinal mTOR signaling pathways after full establishment of a persistent painlike state. We used in vivo electrophysiology, behavioral pharmacology, and immunohistochemistry to show that spinally active mTOR signaling pathways are crucial for the neuronal plasticity and painlike behavior resulting from nerve injury. We achieved this using the rapamycin ester analogue CCI-779 that displays improved water solubility compared with the nonester form of rapamycin, making it more suitable for in vivo studies.^15^

## Methods

All studies were carried out in accordance with the UK Animals (Scientific Procedures) Act 1986 and the International Association for the Study of Pain (IASP) guidelines^41^ and were approved by the United Kingdom Home Office.

### Animals

For all studies, male Sprague Dawley rats (250–280 g for in vivo electrophysiology and 140–160 g for the nerve injury procedure) were used. All rats were supplied (bred in-house) by the Biological Services Unit (BSU), University College London, UK.

### Spinal Nerve Ligation (SNL)

This procedure was completed following a well-established method.^13^ Rats were anesthetized in an induction box with 4% v/v isoflurane in a mixture of nitrous oxide (50% v/v) and oxygen (50% v/v). The isoflurane was reduced to 2.5% (areflexia was maintained). A heating blanket maintained a core temperature of approx 37°C. The left paraspinal muscles were separated from the spinous processes at the lumbar L4–sacral S2 levels. The L6 transverse process was partially removed and the L5 and L6 spinal nerves were isolated and tightly ligated with 6-0 silk threads. This was repeated in sham rats, except for ligation of the spinal nerves. Rats were allowed to recover after closing the wound, prior to sensory testing.

### Behavioral Studies

Rats were tested on days 2, 7, 9, and 14 after surgery to assess the progression of painlike behavior with 1, 6, and 8 g von Frey filaments (Touch Test; Stoelting Co., Dublin, IE) and a cooling stimulus comprising acetone expelled from a modified syringe attached to a piece of tubing. The maximal force of each von Frey filament was applied to the most sensitive part of the ipsilateral and contralateral hind paw a total of 10 times. Acetone was applied to each hind paw a total of 5 times. Stimuli were applied for a total of 2 seconds and each set was separated by at least 3 minutes. Withdrawal responses were confirmed by full lifting of the foot from the stimuli. Difference scores were used to quantify painlike behavior and were calculated as follows: Difference score = number of paw withdrawals from the injured (ipsilateral) side minus number of paw withdrawals from uninjured (contralateral) side. To determine the effect of drugs at the spinal level on painlike behavior, 14 days postsurgery, rats were lightly anesthetized on 2% isoflurane in a mixture of nitrous oxide (50% v/v) and oxygen (50% v/v), after which 20 μL of drug solution containing CCI-779 (250 μM or 12.88 μG in 50 μL saline) was injected through the skin and muscle, into the L5–L6 vertebral interspace, and the rats were allowed to recover for 20 minutes prior to testing. A 20-μL volume has been shown to produce uniform coverage of the spinal cord that is restricted to the sacral and cauda equina levels and extends up to thoracic T13–lumbar L1.^40^ The drug regimen was blinded until the analysis was complete.

### In Vivo Electrophysiology

This procedure followed a well-established protocol.^37^ Rats were anesthetized in an induction box with 4% isoflurane in a mixture of nitrous oxide (66% v/v) and oxygen (33% v/v). The trachea was exposed and isolated and a tracheal cannula was inserted and fastened with 3-0 silk threads. At this stage, the isoflurane was reduced to 2.5% v/v (areflexia was maintained). Rats were then secured in a stereotaxic frame and a rectal probe attached to a heating blanket was used to maintain a core temperature of 37°C.

Vertebral segments were specifically removed to expose L4–L5 of the spinal cord, and muscle and connective tissue from surrounding areas were kept intact to create a well at the exposed spinal cord area into which drug solutions were added. The dura mater was removed to aid drug and electrode penetration. When the set up was complete, the isoflurane was reduced to 1.8% v/v, a level sufficient for anesthesia, while maintaining areflexia. CCI-779 was applied directly onto the exposed spinal cord in a volume of 50 μL (250 nM or 12.88 nG in 50 μL). Behavioral studies used a higher dose of drug (250 μM) compared with electrophysiological studies (250 nM) since 250 μM rapamycin has been shown to be effective in attenuating capsaicin- and nerve injury-induced behavioral hypersensitivity when injected locally into the glabrous skin of the hind paw.^12^ Furthermore, in a previous study, it was found that while a spinal dose of .8 μG of the phosphatdylinositol 3-kinase (PI3K) inhibitor LY294002 was effective in significantly attenuating neuronal excitability in in vivo electrophysiology experiments, a much higher spinal dose of 100 μG was required to attenuate persistent behavioral hypersensitivity.^22^ These differences are likely due to the ease of applying a drug immediately adjacent to the recording electrode in a static system so that lower doses appear more effective for in vivo electrophysiology.

Recordings were obtained with an AC recording system (NeuroLog System, Digitimer; Hertfordshire, UK). An electrode (parylene insulated tungsten microelectrode, 125 μM diameter, 2 MΩ, A-M systems) was manually lowered into the exposed cord (L4–L5) to a depth of 500–1000 μM. This is an area occupied by wide dynamic range (WDR) neurons that respond to innocuous and noxious stimuli. Quantification of neuronal activity was achieved with a 1401 interface and Spike 4 software (Cambridge Electronic Design, Cambridge, UK). An oscilloscope was used to isolate single neurons and a number of stimuli were applied to the receptive field. Mechanical stimuli (von Frey filaments) were applied to the most sensitive part of the receptive field for 10 seconds. This was also the case for thermal stimuli, where increasing heat was applied using a jet of water from a 60-mL syringe attached to a needle.

Electrical stimuli via 2 intradermal stimulating electrodes were used to determine Aß- and C-fiber thresholds based on their latencies to respond to stimuli (Aß-fibers = <20 mS poststimulus; C-fibers = 90–300 mS poststimulus). A train of 16 stimuli (3 times C-fiber threshold at .5 Hz, 2 mS pulse width) was delivered to the receptive field to determine the number of action potentials attributable to Aß-fibers (0–20 mS); Aδ-fibers (20–90 mS); C-fibers (90–300 mS) and postdischarge (300–800 mS) due to the wind-up elicited by repeated stimulation of nociceptive-specific C-fibers. The input (nonpotentiated C-fiber response) and the wind-up (potentiated C-fiber response) were calculated as follows: C-fiber Input = action potentials (90–800 mS) evoked by the first pulse at 3 times C-fiber threshold multiplied by the total number of pulses (16). This represents the theoretical baseline in the absence of wind-up. C-fiber wind-up = total action potentials (90–800 mS) after the 16-train stimulus at 3 times C-fiber threshold minus the input. This represents the excess activity above the theoretical baseline due to wind-up. Only stable cells where 3 consecutive stimulus-evoked responses were within 10% of the previous result for the same test were selected for further pharmacological study, ie, a minimum of 3 control tests were carried out prior to saline or CCI-779 administration. A test comprising electrical, mechanical, and thermal stimuli was carried out every 20 minutes. Saline or CCI-779 was administered 20 minutes prior to the first noncontrol test.

### Immunohisochemistry

Fourteen days postSNL, rats were terminally anesthetized with 1 mL (200 mG) pentobarbital sodium (Euthatal; Merial Animal Health, Essex, UK) intraperitoneally. The rats were transcardially perfused with 200 mL saline with added heparin solution (1000 IU of heparin per l of saline; LEO Laboratories, Buckinghamshire, UK). This was followed by 300 mL per rat of 4% w/v paraformaldehyde (VWR, Leicestershire, UK) solution in .1 M phosphate buffer (PB) mixed with .15% picric acid (Sigma-Aldrich Co., Dorset, UK). Dorsal root ganglia (DRG) from L4, L5, and L6 were removed from both the ipsilateral and contralateral side of the ligations. The lumbar enlargement of the spinal cord was also removed. DRG were postfixed for 2 hours and spinal cord overnight, before being transferred to a cryoprotectant solution containing 30% w/v sucrose in .1 M PB and .01% w/v sodium azide (Sigma) for a minimum of 24 hours. All tissues were embedded in optimal cutting temperature (OCT) compound and rapidly frozen on liquid nitrogen before being stored at –80°C prior to cutting. Transverse sections were cut (20 μM) using a cryostat and mounted on Superfrost Plus slides (Thermo Fisher Scientific Co., Waltham, MA). These slides were then stored at –20°C in a cryoprotectant solution comprising 40% v/v phosphate buffered saline (PBS), 30% v/v ethylene glycerol and 30% v/v glycerol.

### Standard Staining

We probed for the active protein immediately downstream of mTOR, phospho p70S6K. All proteins except phospho p70S6K were probed for using standard staining techniques. Primary antibodies used included rabbit anti-calcitonin gene-related peptide (anti-CGRP, 1:4000; Sigma) and rabbit anti-protein kinase C γ (anti-PKCγ, 1:500, Santa Cruz, Santa Cruz, CA). For all rabbit antibodies, the secondary antibody was goat anti-rabbit cyanine 3 (1:400; Stratech, Newmarket Suffolk, UK). Cryoprotected Slides containing tissue sections were washed several times in PBS. A mixture of 10% v/v normal donkey serum in PBS plus .2% v/v triton X-100 (Sigma) and .1% w/v sodium azide was applied to slides for 30 minutes. The donkey serum was removed and primary antibodies in PBS plus .2% v/v triton X-100 and .1% w/v sodium azide were added to the slides overnight. After several PBS washes, the secondary fluorescent antibody was applied and slides were left in the dark for 3 hours. After several PBS washes, Vectashield (Vector Laboratories, Burlingame, CA) solution was placed onto each slide and a cover slip was positioned over the solution and tissue.

### Tyramide Signal Amplification (phospho p70S6K)

Slides containing tissue sections were immersed overnight in antigen unmasking solution (Vector Laboratories) 1:100 in PBS plus .2% v/v triton X-100 and .1% w/v sodium azide. They were then heated on high power (800 W) for 1 minute 15 seconds. After 30 minutes cooling and several PBS washes, a solution of .3% v/v H_2_O_2_ was applied to the slides for 20 minutes and the slides were again washed several times in PBS. A 10% v/v normal donkey serum in PBS plus .2% v/v triton X-100 (Sigma) and .1% w/v sodium azide was applied to slides for 30 minutes. The serum was removed and primary antibody (rabbit phospho p70S6K Thr389; Cell Signaling Technology, Inc., Danvers, MA) at a concentration of 1:50 in PBS plus .2% v/v triton X-100 and .1% w/v sodium azide was added to the slides overnight. After several PBS washes, the secondary biotinylated antibody (goat anti-rabbit biotin, Vector Laboratories) was applied at a concentration of 1:400 for 1.5 hours. After several PBS washes, slides were incubated with avidin biotin labeled complex (ABC Elite, Vector Laboratories, 1:250 PBS Vectastain solution A plus 1:250 PBS Vectastain solution B) for 30 minutes followed by signal amplification with biotinylated tyramide solution (1:75 PBS for 10 minutes; Perkin Elmer, Waltham, MA). After several PBS washes, extra-avidin fluorescein isothiocyanate (FITC) was used to bind to the biotin attached to the tyramide. Vectashield solution was placed onto each slide and a cover slip was positioned over the solution and tissue.

Sections were viewed under a Carl Zeiss Axioplan 2 fluorescence microscope (Carl Zeiss, Oberkochen, Germany) and images were taken under 10 × or 20 × magnification using the same settings for all sections belonging to the same slide. Postacquisition processing was performed with Adobe Photoshop version 7.0 (Adobe, San Jose, CA) only after all analysis was complete.

### Statistical Analysis

To confirm painlike behavior, comparisons were made between sham and SNL difference scores using the Kruskal-Wallis test with Bonferroni's posttests. The same tests were used to compare the effects of CCI-779 and saline on behavioral hypersensitivity over 2 hours. Electrophysiological raw data are presented as mean ± SEM response. Student's unpaired t-tests were used to compare sham and SNL predrug baseline control neuronal responses with each other, except for graded thermally and mechanically evoked responses, where 2-way ANOVA with Bonferroni posttests were used. In addition, maximal changes in stimulus-evoked responses (positive or negative within the first hour postdrug administration) were analyzed. For drug-induced maximal response changes to electrical, brush, and pinprick stimuli, 1-way ANOVA with Bonferroni's multiple comparisons posttests were used. For maximal changes in graded mechanical and thermal stimuli-evoked responses, 2-way ANOVA with Bonferroni's multiple comparisons posttests were used. For quantification of spinal cord immunohistochemistry, minimums of 3 sections were chosen at random from each rat for each lumbar region (determined with aid of a spinal cord atlas). Regions of interest templates were set (1 set of templates for either the ipsilateral or contralateral side) and maintained for all sections throughout the analysis. Fluorescence levels were determined by calculating the mean grey value using Image J software (National Institutes of Health) and corrected for background levels. All values were then normalized to the side contralateral to the injury. For normalization, all mean grey values were normalized to the single highest mean grey value (100%) of the contralateral side of the corresponding segment resulting in overall contralateral fluorescence levels being less than 100% since the fluorescence levels of each segment represent means across multiple sections. Wilcoxon matched paired tests were then used to compare the normalized ipsilateral mean grey values to the normalized contralateral mean grey values.

## Results

### Confirmation of the Painlike State

**Figure 1:**
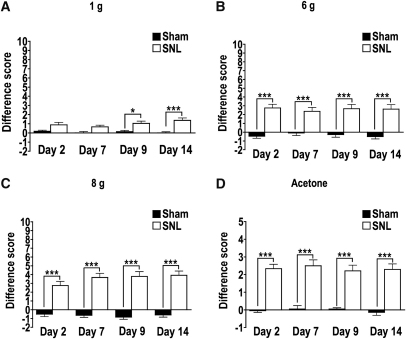
SNL results in painlike behavior. Paired analysis of difference scores show that after SNL (*white*, n = 22), there was a significant increase in paw withdrawals from the injured side versus the uninjured side compared to sham rats (*black*, n = 24). **(A)** For a 1 g stimulus, this increase in behavioral hypersensitivity was most apparent on days 9 and 14. **(B-D)** For a 6- and 8-g stimulus as well as acetone, behavioral hypersensitivity was significantly increased as early as day 2 and maintained up to day 14. ∗*P* < .01. ∗∗∗*P* < .001.

We have shown that SNL produces robust hypersensitivity to innocuous mechanical and cold stimuli of the innervated hind paw from as early as day 2 postsurgery and this is maintained for at least 2 weeks (Figs 1A-D). In fact, this procedure has been found to produce long-lasting hypersensitivity to noxious heat for at least 5 weeks postsurgery and mechanical hypersensitivity at least 10 weeks postsurgery,^13^ perhaps reflecting the longevity of chronic human symptoms in this animal model.

### Effects of CCI-779 on Neuronal and Behavioral Responses From Nerve-Injured Rats

**Figure 2:**
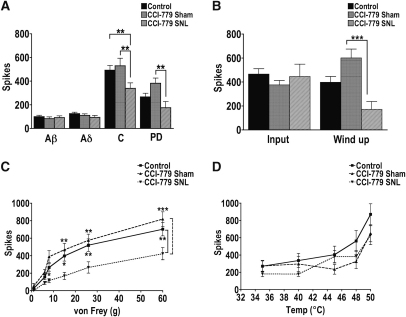
mTOR signaling pathways are more readily inhibited by CCI-779 in SNL rats. **(A)** 250 nM CCI-779 inhibited nociceptive-specific C-fiber–mediated transmission onto WDR neurons from SNL rats (n = 12) compared to sham rats (n = 10) and pooled predrug controls (n = 22). CCI-779 also had a significant inhibitory effect on WDR neuronal postdischarge (PD) compared to sham rats. **(B)** CCI-779 inhibited wind-up, a potentiated response mediated by nociceptive C-fiber activity and a measure of neuronal hyperexcitability from SNL rats compared to sham rats. **(C)** CCI-779 was effective in inhibiting neuronal responses to mechanical stimuli from SNL rats compared to sham rats (8, 15, 26, and 60 g) and pooled predrug controls (15, 26, and 60 g). **(D)** The inhibitory effects of CCI-779 were not extended to thermally evoked responses since there were no differences on thermally evoked responses between pooled predrug controls and CCI-779-treated sham or SNL rats. ∗*P* < .05. ∗∗*P* <.01. ∗∗∗*P* < .001.

It appears that the changes underlying persistent painlike states such as neuropathic pain also change the activity of mTOR signaling pathways to the extent where they are more readily inhibited. In agreement with observations by Suzuki et al,^33^ evoked neuronal predrug responses from SNL animals were equivalent to those in sham animals (Table 1); hence, we pooled SNL and sham predrug responses into 1 control group. CCI-779 had a strong inhibitory effect on specific neuronal responses to electrical and mechanical stimuli from SNL rats compared to predrug control responses and sham rats (Figs 2A-D). When compared to sham rats, C-fiber–mediated transmission onto WDR neurons in SNL rats was significantly inhibited, as were postdischarge and wind-up of WDR neurons (Figs 2A-B), measures of central hypersensitivity mechanisms. There was also significant inhibition of punctate mechanically evoked responses to von Frey filaments 8–60 g (Fig 2C). However, this inhibitory action was highly selective and did not extend to thermally evoked responses, which were unaltered by the drug (Fig 2D), suggesting that in SNL rats, mTOR signaling pathways are particularly important for transmission mediated by mechanical stimuli.

**Figure 3:**
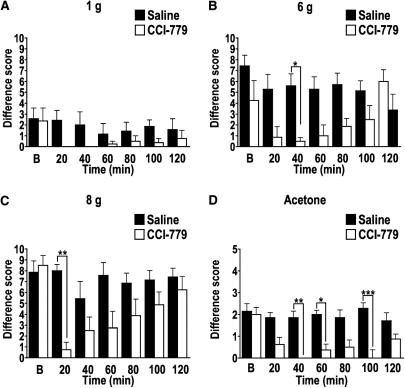
mTOR signaling pathways contribute to behavioral hypersensitivity displayed by SNL rats. **(A)** Paired analysis showed that spinal administration of 250 μM CCI-779 (*white*) resulted in marked (but not significant) attenuation of behavioral hypersensitivity compared to saline (*black*) from baseline (B) levels to a 1 g von Frey filament. Complete (but not significant) inhibition by CCI-779 was observed at 20 and 40 minutes. **(B)** CCI-779 significantly attenuated behavioral hypersensitivity to 6 g at 40 minutes, with a return to baseline levels thereafter. **(C)** CCI-779 significantly attenuated behavioral hypersensitivity to 8 g at 20 minutes, with a return to baseline levels thereafter. **(D)** CCI-779 significantly attenuated behavioral hypersensitivity to acetone at 40, 60, and 100 minutes with some recovery to baseline levels at 120 minutes. ∗*P* < .05. ∗∗*P* < .01. ∗∗∗*P* < .001.

Some of the effects of CCI-779 on neuronal measures were duplicated in awake animals. Behavioral effects were examined in SNL rats at day 14 (Figs 3A-D). Here, the rapid effects on withdrawal responses to mechanical stimuli were much like the effects of the drug seen in the electrophysiological studies. In most cases, maximal inhibition of difference scores occurred at 20 minutes when compared to saline administration. For behavioral withdrawal responses to acetone, inhibition was significant at 20 and 40 minutes (Figs 3A-D). Also, in most cases, inhibition returned to baseline levels, except for withdrawal responses to acetone, where there was a sustained level of inhibition (Fig 3D).

### Distribution of mTOR Signaling Pathways

**Figure 4:**
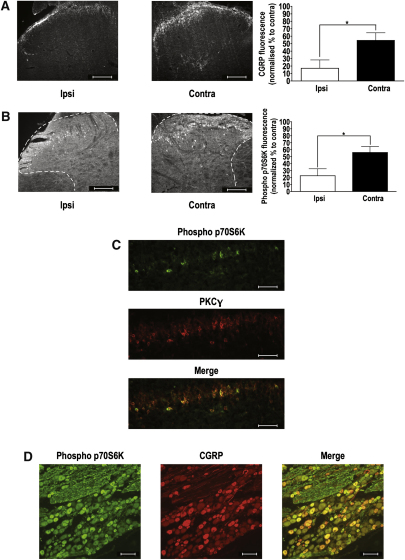
SNL affects CGRP immunoreactivity and mTOR signaling pathways in L5 spinal neurons. **(A)** Observation of the stained tissue sections revealed that immunoreactivity of CGRP in the dorsal horn ipsilateral (Ipsi) to the injury (left) appeared to be lower when compared to the dorsal horn contralateral (contra) to the injury (middle). This was confirmed by a paired analysis of the fluorescence of the ipsilateral versus the contralateral side to the injury (right), thus confirming an expected feature of SNL. Scale bars = 100 μM. **(B)** Likewise, there appeared to be a reduction in phospho p70S6K in the dorsal horn ipsilateral to the injury (left) compared to the side contralateral to the injury (middle), and this was confirmed by paired analysis of the fluorescence of the ipsilateral versus the contralateral side to injury (right). Scale bars = 100 μM. **(C)** Surprisingly, phospho p70S6K was mainly activated in inner lamina II of the dorsal horn of the spinal cord, an area known to be occupied by PKCγ-expressing interneurons. Double labeling spinal cord sections for phospho p70S6K (top) and PKCγ (middle) revealed that the 2 signals were at least partially colocalized, suggesting mTOR signaling pathway activity in a subpopulation of PKCγ interneurons (bottom). Scale bars = 50 μM. **(D)** Double labeling DRG sections for phospho p70S6K (left) and CGRP (middle) revealed that the 2 signals were at least partially colocalized, suggesting that some nociceptive-specific fibers utilize mTOR signaling pathways, at least at the level of the DRG (right). Scale bars = 100 μM. ∗*P* < .05.

**Figure 5:**
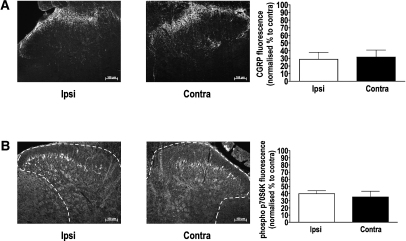
SNL does not affect CGRP and p70S6K immunoreactivity in L4 spinal neurons. **(A)** Observation of the stained tissue sections revealed that immunoreactivity of CGRP in the dorsal horn ipsilateral (Ipsi) to the injury (left) appeared unchanged when compared to the dorsal horn contralateral (contra) to the injury (middle). This was confirmed by a paired analysis of the fluorescence of the ipsilateral versus the contralateral side to the injury (right). **(B)** Likewise, there appeared to be no change in phospho p70S6K in the dorsal horn ipsilateral to the injury (left) compared to the side contralateral to the injury (middle), and this was confirmed by paired analysis of the fluorescence of the ipsilateral versus the contralateral side to injury (right). Scale bars = 100 μM.

Immunohistochemistry showed that SNL resulted in a decrease in immunoreactivity in CGRP in superficial dorsal horn spinal cord regions ipsilateral to the site of injury (Fig 4A). Much like nociceptive CGRP immunostaining, it was found that there was a decrease in immunoreactivity for phospho p70S6K in superficial dorsal horn spinal cord regions corresponding to the ligated L5 and L6 segments ipsilateral to the site of injury (Fig 4B). Interestingly, immunoreactivity for phosho-p70S6K was found to be restricted to the inner layer of lamina II of the dorsal horn and this is an area that is occupied by small interneurons that are immunoreactive for PKCγ.^25^ Double labeling spinal cord sections with both anti-phospho p70S6K and anti-PKCγ revealed that neurons positive for phospho p70S6K appear to form part of a population of neurons that are PKCγ-positive, although not all PKCγ-positive neurons double labeled for phospho p70S6K (Fig 4C). Immunoreactivity for phospho p70S6K was also examined in DRG as an indicator of possible activity in primary peripheral afferent terminals at the level of the spinal cord. Double labeling for phospho p70S6K and CGRP (which is a marker of small, unmyelinated neurons) revealed that mTOR signaling pathways are present in many (but not all) of these neurons at least at the level of the DRG (Fig 4D). Interestingly, we found no reduction in immunoreactivity in the uninjured L4 region, either for CGRP (Fig 5A) or phospho p70S6K (Fig 5B). The positive results from the DRG suggest that mTOR signaling pathways are present in afferents where actions within central terminals could be another substrate for spinally applied modulating agents.

In order to confirm specificity of the secondary goat anti-rabbit IgG for anti-phospho p70S6K, control experiments were carried out whereby the primary antibody (anti-phospho p70S6K) was omitted from the protocol. Here there was an absence of immunoreactive signal (data not shown). In addition, although the secondary antibodies used for double labeling were both produced in rabbit, amplification of the p70S6K signal results in a complex that is only recognized by a biotinylated secondary antibody. Although intuitively, one would expect there to be some cross-reactivity between the secondary antibodies, it has been shown that when a primary antibody is used at a concentration that can only be detected by a fluorescent amplification method (we saw no detection without the amplification method), another primary antibody, raised in the same host species, can be used and demonstrated with a different flurochrome in subsequent conventional immunostaining of the same section since cross-reactivity is not an issue.^3,10,38^

## Discussion

In recent years, there has been growing interest in the roles of mTOR signaling pathways, and particularly, their roles in neuronal plasticity. Neuronal plasticity is a widely accepted feature of many persistent pain states observed clinically. Here, we provide convincing evidence using in vivo electrophysiology, behavioral pharmacology, and immunohistochemistry that these pathways contribute to neuronal plasticity and behavioral hypersensitivity in a model of chronic neuropathic pain. The list of neurotransmitters, receptors, ion channels, and genes that have been identified as key modulators of persistent and chronic pain states is extensive. Perhaps most interesting is that many neurotransmitters, receptors, and ion channels implicated in persistent and chronic pain states engage mTOR signaling pathways^11,34^ that may also regulate the translation of specific pain genes. Thus, mTOR signaling pathways at the level of mTOR may represent a convergence point of many nociceptive signals, which is an intriguing prospect for future basic and clinical studies.

Our present studies show that nerve injury induces changes at the spinal level that result in neuronal plasticity and behavioral hypersensitivity and that these changes depend upon activity of mTOR signaling pathways. CCI-779 modulated responses of spinal cord neurons in the neuropathic model in a selective manner since specific measures of central hypersensitivity such as wind-up and postdischarge were reduced, as was the C-fiber—evoked activity. With natural stimuli, there was attenuation of mechanically evoked activity with no effect at all on thermally evoked responses. These findings were corroborated by the drug being effective on mechanical (and cooling) responses in SNL animals.

Of particular interest is that the efficacy of CCI-779 is specifically increased after direct nerve injury. The effectiveness of the anti-neuropathy drug gabapentin (GBP) in attenuating neuronal responses of SNL rats^32^ is similar to what we observed here although the agents have very different modes of action. Both drugs were weakly effective or ineffective on neuronal responses in the absence of nerve injury.

Our previous studies showed that selective mTOR inhibitors such as rapamycin attenuate the neuronal plasticity and behavioral hypersensitivity associated with formalin by inhibiting mRNA translation since they exert the same effects as the global mRNA translation inhibitor anisomycin.^1^ Therefore, we are confident that CCI-779 acts specifically by inhibiting mTOR signaling pathways. Although not investigated further in this study, we are also confident that the results produced in our electrophysiological and behavioral data reflect a reduction in p70S6K activity. Our behavioral dose of 250 μM CCI-779 is equivalent to 12.88 μG per 50 μL dose, and Codeluppi et al^6^ and Jimenez-Diaz et al^12^ show that 1 mg/kg and 12.5 μG per 50 μL dose respectively of rapamycin attenuate the phosphorylation of p70S6K. Perhaps more relevant, is a study by Podsypanina et al^24^ that shows that a high CCI-779 dose of 20 mg/kg attenuated phospho p70S6K activity in endogenous tumors.

The relatively restricted neuronal localization of activated phospho p70S6K is also of interest because these neurons double label for PKCγ, indicating that a proportion of spinal neurons, which contain active mTOR signaling pathways, also express PKCγ. PKCγ is important because it has been shown that mice that lack the PKCγ isoenzyme fail to develop neuropathic painlike syndromes and the neurochemical changes that occur in the spinal cord after nerve injury.^18^ Besides being located in inner lamina II, PKCγ interneurons are also present in lamina III, with some weak immunoreactivity in lamina I and the outer layer of lamina II. Most PKCγ-positive neurons are not GABA or μ-opioid receptor-immunoreactive,^25^ substantiating their roles as excitatory interneurons that have roles in the maintenance of persistent painlike states. Although we failed to detect immunoreactivity for phospho p70S6K in deeper lamina of the dorsal horn of the spinal cord, it is not surprising that we still observed changes in neuronal output from WDR neurons since it has been previously shown that ablating lamina I superficial (and to a lesser extent, lamina III) neurons will affect neuronal excitability of deeper WDR neurons.^31,32^ Importantly, responses from WDR neurons to a wide range of innocuous and noxious stimuli and their ability to wind up to electrical stimulation are unique to this class of neurons and the information we obtain from them can provide an important measure of intrinsic excitability of the spinal cord.

Initially, it was widely accepted that PKCγ interneurons in the spinal cord received their input from unmyelinated, nonpeptidergic, IB4-positive nociceptors.^29^ However, in a recent study, it has been shown that the terminal field of IB4-positive neurons in fact lies dorsal to that of PKCγ interneurons.^19^ In contrast, medium diameter and large diameter myelinated afferents that are generally associated with innocuous signal transmission, extensively overlap with PKCγ interneurons. Furthermore, PKCγ interneurons are specifically activated by innocuous input generated by rotarod-induced locomotion. These studies suggest that A-fiber—mediated transmission should be affected by CCI-779 and also that PKCγ interneurons may not play an important role in our model of chronic neuropathic pain. However, when assessing the effects of CCI-779 on A-fiber—mediated transmission, we saw no significant effects. This may be due the fact that A-fibers were stimulated at 3 times the C-fiber threshold during an electrical stimulus train and therefore that all A-fiber—mediated changes were masked. Nevertheless, some of the responses to lower von Frey filaments (<15 g) are likely A-fiber—mediated and these responses were amenable to inhibition by CCI-779.

Despite the fact that we see immunoreactivity for phospho p70S6K in DRG, it is unlikely that CCI-779 exerts its effects on primary afferent terminals but rather, exerts its action specifically on spinal neurons since Geranton et al^7^ have recently reported that mTOR signaling pathways do not extend beyond the dorsal root entry zone and double staining spinal cord sections for phospho mTOR and synaptophysin, synapsin, or vesicular glutamate transporter 1 (VGLUT1) did not show localization of mTOR in axon terminals. Regardless of the specific roles of PKCγ interneurons, they must undoubtedly be important in sensory processing in the spinal cord since we show that phospho p70S6K immunoreactivity of PKCγ interneurons and the neuronal and behavioral phenotype are all altered after nerve injury. Our data shows an overall inhibitory effect of CCI-779 on C-fiber–mediated transmission onto WDR neurons and therefore noxious stimulus-evoked responses.

Our finding that SNL results in a decrease in immunoreactivity for phospho p70S6K in the corresponding lumbar spinal segment (L5) is of particular interest because intuitively, based on previous studies, particularly concerning neuronal activity in the brain, a condition like neuropathy would be expected to result in an increase in phospho p70S6K immunoreactivity.^5,21,35,36^ Intriguingly, one-third of lamina II interneurons receive simultaneous monosynaptic inputs from 2 to 4 different segmental roots.^23^ This model of organization, where substantia gelatinosia spinal neurons at L4 receive major monosynaptic input from L4-L6 roots, suggests that the reduction in L5 phospho p70S6K and CGRP immunoreactivity after SNL should also be observed in L4 (even though at this level, spinal nerves are not ligated) and that neurons within L4 will have reduced responses. However, we find that phospho p70S6K, CGRP immunoreactivity are actually maintained in what should be partly denervated L4 of SNL animals. We also find no difference in neuronal responses between sham and SNL animals, where in both cases, evoked neuronal activity is recorded from L4 and L5 segments. These results can be explained by the following hypotheses and conclusions.

1. **Immunohistochemistry**. Ligation of the L5 and L6 spinal nerves result in loss of input to the dorsal horn of L4 as well as L5 and L6, corresponding with a reduction in the level of immunohistochemical markers for p70S6K and CGRP in L5. This should also correspond with a reduction in the level of immunohistochemical markers for p70S6K and CGRP in L4. Since this is not the case, we propose that there is an upregulation of p70S6K phosphorylation and CGRP in L4 of the spinal cord that masks the deficit of these markers.
2. **Electrophysiology**. Ligation of the L5 and L6 spinal nerves results in loss of input to the dorsal horn of L4 as well as L5 and L6 which should correspond with a reduction in the excitability of WDR neurons recorded from L4 and L5. Although we did not distinguish between L5 and L4 neuronal responses in individual animals, the fact that we see no difference between responses from sham and neuropathic rats suggests that there may be increase an excitability of L4 WDR neurons and afferents in neuropathic rats which increases the excitability of the neighboring L5 segment so that WDR neurons in L4 and L5 are also more excitable. This increase in neuronal excitability in neuropathic rats thus masks the deficit of L5 input. In agreement with these statements, previous studies have shown that spinal neurons with connections to the periphery expand their receptive fields after the same nerve ligation, so that a given spatial stimulus will activate more spinal neurons compared to sham animals, a likely substrate for the enhanced nociception present in this model.^30^

There may well be local changes in mTOR activity that were not detected with our methods, such as changes restricted to specific synapses. However, in accordance with our interpretation of our data is ample evidence that abnormal neuronal activity from uninjured segments of the spinal cord contribute to the neuronal and behavioral phenotypes associated with persistent painlike states. Specifically, the marker for damaged neurons-activating transcription factor 3 (ATF3) is enhanced in L4 spinal neurons even though L5 spinal nerves are ligated.^28^ In injured L5 DRG, SNL has been found to induce the activation of multiple mitogen-activated protein kinases (MAPKs) in different populations of DRG neurons whereas in the uninjured L4 DRG, the L5 SNL induces p38 activation in tyrosine kinase A-expressing small- to medium-diameter neurons.^20^ Furthermore, L5 SNL has been shown to cause extensive changes in the electrophysiological profile of large- and medium-sized, but not small-sized somata of intact L4 as well as axotomized L5 DRG neurons after L5 spinal nerve ligation.^17^ In addition, transient receptor potential V1 (TRPV1) of the A-fiber somata has been shown to increase in the uninjured L4 DRG compared to controls,^9^ and L4 dorsal rhizotomy performed 7 days after the L5 SNL has been shown to result in an immediate reversal of low mechanical withdrawal thresholds back to baseline values.^16^

Interestingly, CGRP nociceptors were immunoreactive for phospho p70S6K in DRG. This finding differs from Geranton et al^7^ and Jimenez-Diaz et al,^12^ who found that within the glabrous skin of the hind paw, the majority of phospho mTOR and mTOR staining was found to coexist with N52 immunoreactivity in A-fibers. This apparent contradiction appears to be due to a mismatch between immunoreactivity in DRG and immunoreactivity at peripheral afferent terminals. In support of this is the finding by Geranton et al^7^ that phospho mTOR signaling pathways do not extend beyond the dorsal root entry zone. It is important to note that Jimenez-Diaz et al^12^ did find that rapamycin affected thresholds of some C-fibers, suggesting that mTOR signaling pathways are also important in C-fiber–mediated transmission. Furthermore, the mTOR binding protein raptor was found to be expressed in a small proportion of N52-negative fibers and more recently, in a small population of peripherin (nociceptor marker)-positive neurons. In these most recent studies by Geranton et al,^7^ it is reported that signaling pathways involving phospho mTOR are localized in lamina I and lamina III. However, we find phospho mTOR labeling in inner lamina II where activity is localized with PKCγ interneurons. It is likely that our model of neuropathic pain, whereby we ligate spinal nerves L5 and L6 and euthanize and perfuse on day 14 postsurgery, results in neuron-specific changes in mTOR signaling in the entire affected region of the spinal cord that would not be seen in uninjured animals like those used by Geranton et al for their immunohistochemistry.^7^

Our immunohistochemistry data indicates that active mTOR signaling at the spinal level contributes to the neuronal plasticity and behavioral hypersensitivity associated with persistent painlike states. We hypothesize that CCI-779 acts on PKCγ-positive spinal interneurons, causing a reduction in nociceptive signals transmitted by C-fibers. The fact that there is some degree of restriction in terms of the expression of this pathway probably confers selective modulation of mechanical responses and hypersensitivity without altering thermal sensory processing.

In our previous study,^1^ we showed that rapamycin produces inhibitory effects on C-fiber activity, mechanically and thermally evoked responses in naive animals. We also showed that spinally administered rapamycin attenuates formalin-induced hyperexcitability, a finding which was first shown by Price et al.^27^ In this study, we did not observe inhibitory effects by CCI-779 in sham animals. Given that CCI-779 is an analogue of rapamycin, we would expect to see similar effects of CCI-779 on neuronal responses in sham animals. However, it is likely that although CCI-779 shows improved water solubility, subtle changes in pharmacokinetics, which are dependent upon the solution into which the compounds are diluted, may mean that the inhibitory effects are altered at least in nonpathological states, such that 250 nM rapamycin is more efficacious than 250 nM CCI-779, at least in our hands. Importantly, CCI-779 exerts strong inhibitory effects on SNL-induced behavioral hypersensitivity compared to saline.

Particularly evident in the behavioral studies was that attenuation of behavioral hypersensitivity was only temporarily inhibited after administration of CCI-779. This rapid yet temporary maximal effect of CCI-779 indicates that at least in this pain model, mTOR signaling pathways may be continuously active and that their continuous activation rapidly regulates neuronal activity and behavior. Such rapid signaling, which has a direct effect on neuronal function, is not a novel concept. In acute brain slices it has been shown that that the induction of protein synthesis-dependent hippocampal LTP increases the local expression of elongation factor 1A (eEF1A) in dendrites that are severed from their cell bodies before stimulation. This effect is blocked by rapamycin, indicating that the increase in eEF1A expression is mediated by mTOR pathway. Remarkably, eEF1A levels increase within 5 minutes after stimulation in a translation-dependent manner, and this effect remains stable for 3 hours.^36^

## Conclusions

By focusing on subcellular targets that are implicated in pain, the hope is that we will be able to produce selective drug treatments that are more effective and exert fewer side effects than what is currently available. These studies, along with our previous studies,^1^ confirm that mTOR signaling pathways are important in nociception and persistent painlike states. The widespread distribution of these pathways as well as their numerous cellular effects that include mediating the immune response means that the therapeutic potential of CCI-779 remains in question. However, these studies reveal that rapid, continuous mTOR signaling at the spinal level is an integral element of nociception and the maintenance of persistent painlike states involving central sensitization.

## Figures and Tables

**Table 1:** Differences Between Baseline WDR Neurone Responses From Sham and SNL Rats

	Sham (n = 10)	SNL (n = 12)
Aß-fiber spikes	87 ± 16	111 ± 16
Aδ-fiber spikes	112 ± 19	136 ± 15
C-fiber spikes	461 ± 55	531 ± 57
Post-discharge spikes	293 ± 33	245 ± 49
Input spikes	480 ± 64	464 ± 64
Wind up spikes	467 ± 46	344 ± 82
Brush spikes	572 ± 89	445 ± 52
Pin spikes	400 ± 75	311 ± 59
1 g spikes	31 ± 17	21 ± 10
6 g spikes	195 ± 46	125 ± 44
8 g spikes	300 ± 60	237 ± 65
15 g spikes	437 ± 83	364 ± 84
26 g spikes	570 ± 101	479 ± 102
60 g spikes	782 ± 118	637 ± 100
35°C spikes	342 ± 104	192 ± 47
40°C spikes	427 ± 143	234 ± 61
45°C spikes	444 ± 167	359 ± 99
48°C spikes	610 ± 204	509 ± 278
50°C spikes	975 ± 208	754 ± 118

NOTE. The table shows predrug control responses of WDR neurones from sham and SNL rats. For electrically evoked responses, a train of 16 pulses at 3 times C-fiber threshold (.5 Hz, 2-mS pulse width) was applied to the corresponding hind paw. For mechanically evoked responses, von Frey filaments of ascending force were applied to the corresponding receptive field for 10 seconds. For thermally evoked responses, a small water jet was applied to the corresponding receptive field for 10 seconds. All data are expressed as raw mean values ± SEM. There were no significant differences between WDR neurone responses from sham or SNL rats prior to CCI-779 administration.
